# The relationship between trait mindfulness and psychotic-like experiences in a brief AI-generated music listening context: the roles of presence, perceived interactivity, and emotional arousal

**DOI:** 10.3389/fpsyt.2026.1859243

**Published:** 2026-07-28

**Authors:** Chenghan Zhang, Hao Huang, Guotao Wu, Mengke Luo

**Affiliations:** 1 School of Music of Nanjing University of the Arts, Nanjing, China; 2 Industrial Digital Research Centre, Huainan Normal University, Huainan, China

**Keywords:** AI music, arousal, mindfulness, perceived interactivity, presence, psychotic-like experiences, structural equation modeling

## Abstract

**Background and objective:**

Within the interdisciplinary field of cyberpsychology and mental health, trait mindfulness has been associated with lower levels of subclinical anomalous symptoms, such as Psychotic-Like Experiences (PLEs), has gained increasing attention. However, in the context of daily digital human-computer interactions (e.g., listening to AI-generated music), the specific pathways through which Mindfulness operates (the involvement with Presence and Perceived Interactivity) and the boundary conditions of physiological arousal, remain to be clarified. This study aims to explore the direct predictive relationship between mindfulness and individuals’ PLEs, and to investigate the multipath effect of brief AI-generated music listening context (with Presence and Perceived Interactivity), along with the moderating effect of Arousal.

**Methods:**

With a cross-sectional survey design, self-reported multimodal data were collected from 527 Chinese participants. Structural equation modeling (SEM) was conducted using Mplus 8.3 to empirically test the main effects (path coefficients) of the theoretical hypotheses and the moderation model.

**Results:**

Both the measurement and structural models demonstrated good fit. The path analysis results indicated that: (1) trait trait mindfulness was significantly and negatively associated with PLEs (p < 0.001); (2) regarding the main effect paths of brief AI-generated music listening context, mindfulness significantly and positively predict individuals’ Presence and Perceived Interactivity, while both significantly and negatively predict PLEs; (3) Arousal played a significant moderating role in the relationship between mindfulness and brief AI-generated music listening context, exhibiting a synergistic enhancement effect. Higher levels of Arousal significantly amplified the positive prediction of Mindfulness on both Presence and Perceived Interactivity (p < 0.01).

**Conclusions:**

With the help the SEM, this study maps out the underlying multipath network through which mindfulness is associated with lower PLEs within a brief AI-generated music listening context. The observed associations suggest that presence and perceived interactivity may function as pivotal correlational nodes relevant to mental health correlates, while these results also nuance classic cognitive load assumptions by indicating a potential synergistic association between trait mindfulness and emotional arousal. These results provide a solid empirical foundation and prospective insights, for the mental health-oriented design of AI music products, such as the immersive acoustic environment construction and dynamic, arousal-based interaction recommendations.

## 1 Introduction

Nowadays, the escalating psychological disorders highlights the direct link between subclinical symptoms and clinical schizophrenia, further exacerbating the dual challenges of resource shortages in global public mental health and a mounting disease burden (1). This increasingly severe, latent psychological crisis demands highly efficient and low-barrier preventive interventions, to effectively halt the continuous deterioration of individuals’ psychological states (2). In the modern high-stress societies, the general public is confronting the dual pressures of cognitive overload and intensified psychological alienation. According to the data from cross-national epidemiological survey, up to 5% to 8% of the global non-clinical population report having frequently encountered Psychotic-Like Experiences (PLEs) (3), with even higher proportion among younger groups. Consequently, the international public health community has recognized the early intervention for PLEs as a crucial component of mental illness prevention strategies, which has been integrated into a broader framework of universal mental health promotion, as well as the psychosocial service systems (4). Against this backdrop, Mindfulness-based interventions, especially those empowered by cutting-edge AI music technologies, have emerged as a frontier strategy and a critical pathway for buffering Psychotic-Like Experiences and rebuilding individuals’ psychological resilience (Xiang, 2025).

However, achieving these ambitious goals of early prevention and intervention relies heavily on the positive reshaping of core psychological traits, because Mindfulness serves as a critical micro-psychological mechanism for regulating maladaptive cognitive loops, exerting a substantial protective effect on mental health (1). In recent years, with the deep penetration of next-generation digital technologies, including generative artificial intelligence, affective computing, and machine learning, customized AI music (AI-generated music) (5) has emerged as a crucial catalyst for enhancing the efficacy of Mindfulness-based interventions. It facilitates the digital transition of traditional meditation paradigms and significantly enhances the psychological compliance and experiential depth of the intervention recipients (6). brief AI-generated music listening contexts utilize adaptive algorithms to generate dynamic soundscapes tailored to individuals’ real-time physiological rhythms and emotional states (7). Rather than being a mere overlay of background audio, it represents a systematic process of psychological transformation that integrates external sensory stimuli, reorganizes internal attention allocation, and facilitates pathological cognitive defusion (8). Multiple cutting-edge medical consensuses, including the global wave of Digital Therapeutics (DTx) (9), have identified the synergistic innovation between intelligent sensory technologies and evidence-based psychological interventions as a priority research agenda (10). This further consolidates the bridging role of digital assistive technologies in the early prevention and treatment of psychosis and in sustainable mental health management (11).

In line with the global trajectory of Digital Therapeutics (DTx) (12), more and more interdisciplinary research have examined the potential of Mindfulness-based interventions supported by AI music to alleviate Psychotic-Like Experiences (PLEs) (13). These studies specifically focus on the diverse pathways through which intelligent soundscape technologies can reshape individuals’ cognitive and perceptual practices (14). Empirical evidence largely suggests that technology-enhanced Mindfulness restructures individuals’ attention allocation and emotions processing. This restructuring effectively eases the cognitive load required to suppress maladaptive thoughts, thereby buffering psychopathological symptoms (15). Recent studies indicate that dynamically adaptive digital soundscapes allow individuals to navigate internal psychological fluctuations with greater flexibility (16). With profoundly relaxing experience and the non-judgmental awareness, these auditory environments help address the effect of aberrant salience through a dual-pathway mechanism: top-down cognitive defusion and bottom-up sensory regulation (17). Highly immersive audio interventions have also shown their abilities to significantly elevate interoceptive awareness, thereby increasing tolerance for intrusive delusions and improving the quality of emotional self-regulation (18). Such highly customized musical spaces can effectively mitigate feelings of dissociation and paranoid rumination. Simultaneously, they provide a safer, more enveloping external support, which is highly conducive to psychological recovery and Mindfulness-based practice (19). Empowered by spatial audio and generative algorithms, AI music further bolsters the efficacy of traits Mindfulness in warding off severe psychological crises (20). It achieves this comprehensive enhancement through cutting-edge dimensions, specifically by inducing a profound sense of Presence and fostering deep Perceived interactivity between the user and the system (21).

With these notable advancements above, existing research on digital Mindfulness-based interventions remains largely confined to the well-known aspects, such as technical usability evaluation or superficial improvements observation in global symptoms (22), neglecting the critical issue of the internal coherence between underlying sensory experiences and higher-order cognitive reconstruction (67). Based on the concept of Embodied Cognition and Cognitive Load Theory, there is a problematic phenomenon emerging amidst fervent embrace of cutting-edge “AI-generated” in the digital healthcare market. Many users tend to treat highly immersive AI music as a mere tool for passive acoustic distraction, seeking only temporary emotional relief (23). Consequently, they often fail to achieve deep psychological spatial anchoring for a profound Presence, and the necessary emotional resonance required for Perceived interactivity (24). Beside this issue, the efficiency of these sensory input transmission into deep psychological experiences is heavily constrained by individuals’ autonomic nervous system baseline. When individuals operate under abnormal Arousal, internal cognitive noise can directly obstruct the intended empowerment of technologies (25). “Perceptual-interactive decoupling”, as a pronounced disconnect between physical exposure and psychological investment, may lead to a failure of the cognitive regulatory resources reallocation within the AI system, ultimately undermining the inherent capacity of Mindfulness to buffer psychopathological symptoms (26). Therefore, whether the traits Mindfulness supported AI music could effectively alleviate Psychotic-Like Experiences (PLEs) not only depends on the chained linkage of Presence and Perceived interactivity, but also on the moderating role of underlying physiological Arousal, which functions as a substantive boundary condition (27).

To bridge gaps in the existing research, this study categorizes the digital processing of mindfulness supported by brief AI-generated music listening contexts into two progressive core dimensions (28): spatial psychological anchoring (Presence) and relational emotional resonance (Perceived Interactivity) (29). It further frames the chained transmission of “mindfulness-presence-interactivity” as an effective intervention mechanism for buffering psychopathological symptoms. Drawing upon empirical data from China, this study offers three primary contributions. First, by exploring the correlational link between trait mindfulness and psychotic-like experiences (PLEs), this study extends prior scholarship that framed mindfulness only as a standalone meditation construct separated from technological scenarios (30). This correlational pattern suggests that well-designed AI music auditory environments may align in ecologically meaningful ways with individuals’ dispositional awareness capacities, which offers a tentative viewpoint relevant to the design of future digital mental health schemes. (31). Second, moving beyond direct effects, the investigation reveals the chained mediation mechanism through which traits mindfulness alleviate PLEs. Mindfulness initially induces a high degree of Presence, which subsequently transforms into Perceived Interactivity with the digital system (32). These findings not only open the “black box” of digital therapeutics, but also illustrate how physical audio exposure transforms into higher-order cognitive reconstruction. This study deepens our understanding of the underlying psychological mechanics and provides the mainstream clinical psychology with a precise implementation framework for integrating human-computer interaction practices in the intelligent era (33). Third, through a systematic analysis of boundary conditions, this study evaluates the comprehensive moderating effect of individuals’ underlying physiological and emotional baseline, Arousal, on these complex pathways. This variable establishes a multidimensional analytical framework spanning cognitive, perceptual, and physiological levels. The excessive Arousal reflects individuals’ risky exposure to internal cognitive noise and emotional dysregulation (34); it not only diminishes the efficiency with which mindfulness transforms into immersive Presence and Perceived Interactivity, but also directly constrains the ultimate buffering effect of traits mindfulness on Psychotic-Like Experiences (35). By integrating this heterogeneity in underlying physiological mechanisms, this study provides a more refined theoretical elucidation of the boundary conditions governing digitally driven mental rehabilitation, yielding significant clinical value and interdisciplinary implications.

## 2 Literature review and hypotheses

### 2.1 Mindfulness and psychotic-like experiences

Mindfulness refers to individuals’ intentional self-regulatory process anchoring their attention to the present moment, while maintaining a non-judgmental awareness to the emerging internal and external psychological stimuli (36). From the perspective of metacognition and information processing, traits Mindfulness profoundly enhances individuals’ capacity to allocate and utilize limited resources for cognitive controlling more efficiently (37). Specifically, the non-reactive awareness of immediate internal psychological events, including intrusive thoughts and anomalous perceptions, enables individuals to monitor the flow of their attention with greater precision (38). Decreased pathological rumination conserves cognitive bandwidth and mitigates the secondary psychological distress triggered by paranoid thoughts (39). Such traits further promote cognitive defusion between sensory experiences and higher-order cognitive appraisals. Particularly when individuals face with ambiguous or threatening anomalous perceptions, this trait disrupts the erroneous fusion of subjective fantasy with objective reality, by reinforcing the objective desensitizations, to fundamentally recognize that “thoughts are just thoughts” (40). The robust synergistic effect between attentional redirection and the non-judgmental awareness empowers individuals to better integrate internal sensory anomalies with external authentic feedback (41). This integration heightens the conversion efficiency of emotion regulation mechanisms, ultimately providing an effective blockade against the deterioration of subclinical symptoms into overt psychopathology (Wang et al., 2025). The processing reconstruction at the metacognitive level embeds non-judgmental awareness directly into daily stress-response chains, accelerating the iteration of adaptive emotions (42), which curtails maladaptive cognitive loops and attenuates the brain’s aberrant salience to irrelevant stimuli, leading to a marked decrease in the frequency and intensity of Psychotic-Like Experiences (43). Drawing upon the aforementioned theoretical deductions and empirical observations, this study proposes the following hypothesis:

H1: Trait mindfulness is significantly and negatively associated with individuals’ PLEs.

### 2.2 Mindfulness, presence, and perceived interactivity

By heightening individuals’ “attentional and sensory transparency”, Mindfulness exerts a dual promotion effect on the dimensions of digital experience within brief AI-generated music listening contexts (44). Beyond reshaping neuropsychological architectures and emotion-regulation paradigms, Mindfulness establishes a real-time perceptual flow between the individual and present-moment acoustic stimuli, thereby drastically increasing the transparency of both internal experiences and external inputs (45). A critical driver of this promotion effect lies in the high-level congruence between physical exposure and psychological investment (46). Particularly among individuals with high-level mindfulness in interventions, this congruence generates a unique “cognitive processing premium” (47). By leveraging psychological traits such as non-judgmental awareness and present-moment anchoring, individuals can achieve real-time perceptual traceability of dynamic AI soundscapes (48), crafting a high-fidelity mapping between objective audio input and subjective psychological anchoring (49). This high-fidelity sensory mapping substantially lowers the information validation costs inherent in the brain’s predictive coding processes, by permitting the executive control network (ECN) seamless access to unbiased auditory data (21). Serving as a vital informational mediator within the human cognitive system, metacognitive monitoring utilizes its awareness mechanisms to interpret the emotional cues embedded in AI music, so as to evaluate the progress of the interaction, consequently refining appraisals of aesthetic value and safety within the digital spaces (11). This refinement optimizes information transmission across multisensory channels, captures greater bottom-up attention, and ultimately alleviates cognitive resource constraints at the working memory level, fostering a profound engagement with the digital experience (50).

Since the metacognitive system demonstrates a profound anchoring interest in the ongoing stream of AI music, individuals are subjected to greater endogenous awareness constraints, which effectively suppresses brain’s “cognitive opportunism” (manifested as mind-wandering or paranoid rumination), and alleviates the psychological detachment induced by task-unrelated thoughts (51). Guided by this sustained mindful scrutiny, individuals are more likely to preserve the alignment between sensory reception and psychological processing, optimizing their attentional resources allocation, and ultimately transforming the acoustic benefits of digital music into a substantive therapeutic experience (52). Through this transmission mechanism, the digital experience undergoes a marked elevation in both its “spatial depth” and “relational warmth” (53). From the spatial perspective, the cognitive constraints alleviation heightens the intensity of psychological anchoring within the music-constructed virtual environment, manifesting as a profound Presence (54). From the relational perspective, amplified metacognitive monitoring inhibits mechanical desensitized reactions to AI stimuli and facilitate a shift toward deep Perceived Interactivity, which is a state fundamentally underpinned by emotional resonance and dynamic feedback (55). Guided by this logical reasoning, this study proposes the following hypotheses:

H2: Mindfulness significantly and positively predicts individuals’ Presence within brief AI-generated music listening contexts.

H3: Mindfulness significantly and positively predicts individuals’ Perceived Interactivity within brief AI-generated music listening contexts.

### 2.3 Presence and psychotic-like experiences

By elevating individuals’ “reality-testing clarity”, AI music-induced Presence plays a dual suppressive role against Psychotic-Like Experiences (PLEs) (56). Beyond reshaping auditory environments and emotional baselines, the profound Presence establishes a real-time perceptual flow between individuals and the secure soundscape, drastically increasing the transparency of the boundary between internal self and external reality (57). By generating the so-called unique “cognitive containment premium” within digital health interventions, the high congruence between physical auditory exposure and psychological spatial anchoring serves as the pivotal driver behind this buffering effect of Presence (54). Through the cutting-edge digital technologies utilization, including spatial audio and adaptive generative algorithms, individuals can attain real-time embodied traceability of the musical context at the psychological level, thereby forging a robust mapping between the objective digital soundscape and a subjective secure attachment (Reali, 2024). Such high-fidelity spatial anchoring substantially reduces brain’s cognitive validation costs with ambiguous or hallucinatory stimuli, specifically by granting bottom-up sensory networks to prioritize access to absolute safe, non-threatening auditory data (58). Serving as a critical informational mediator between the human mind and the external environment, embodied feeling leverages its spatiotemporal anchoring mechanisms to interpret safety cues within the digital space and gauge relaxation progress, therefore correcting the flawed appraisals of latent external hostility by the schizophrenic cognitive system (59). It optimizes the benign attention allocation and recruits additional exogenous cognitive resources, alleviating the working memory capacity constraints induced by paranoid internal depletion and fostering a adaptive re-engagement with the real world (60).

Individuals’ sensory system exhibits intense interest dwelling within the AI music space, their cognitive system is subjected to a stronger “here and now” anchoring pressure, which could effectively constraint the “cognitive opportunism” (manifested as unconscious paranoid wandering or hallucinatory constructions) among high-risk individuals, mitigating proxy symptoms like depersonalization and derealization (61). Under the scrutiny of intense psychological dwelling, individuals are more apt to maintain the alignment between sensory reception and objective reality, allocating their limited attentional resources with far greater circumspection, and converting the enveloping advantages of AI acoustics into substantive psychological resilience (62). Through this precise transmission mechanism, both the frequency and severity of Psychotic-Like Experiences (PLEs) are markedly attenuated. From the perspective of frequency, the cognitive constraints alleviation undermines the neural substrate responsible for generating aberrant salience, thereby curbing the rate of delusional thoughts generation (63). From the perspective of severity, reinforced spatiotemporal anchoring (profound Presence) effectively suppresses opportunistic pathological rumination, and facilitates individuals’ transition toward a healthy cognitive state underpinned by authentic psychological safety and reality attachment (64). Guided by above logical deduction, this study proposes the following hypothesis:

H4: Presence significantly and negatively predicts individuals’ Psychotic-Like Experiences (PLEs).

### 2.4 Presence and perceived interactivity

Within the phenomenological framework of human-computer interaction, presence, particularly spatial presence, refers to a user’s subjective sense of being psychologically located in a mediated environment (65). In the context of brief AI-generated music listening context, presence describes the extent to which individuals feel immersed in, surrounded by, and attentively anchored within the AI-generated acoustic environment (66). Rather than treating music as a distant external stimulus, individuals with a high level of presence experience the digital soundscape as a psychologically meaningful space in which their attention and perception are situated. Perceived interactivity, in contrast, refers to users’ subjective assessment of the extent to which a digital system provides responsive, reciprocal, and controllable interaction. It is conceptually different from objective interactivity, which concerns the technical features or interactive functions provided by a system (67). Perceived interactivity emphasizes the user’s psychological judgment that the system is responsive to their input, preferences, emotional state, or real-time engagement. In the present study, perceived interactivity refers to the degree to which individuals perceive AI-generated music as adaptive, responsive, and capable of engaging in a form of personalized interaction.

Based on these definitions, presence serves as a basic experiential condition for the formation of perceived interactivity. When individuals achieve a stronger sense of spatial anchoring within the AI music acoustic envelope, they may more effectively detach their cognitive resources from external physical distractions and reallocate attention to the digital soundscape (68). This heightened psychological reality can increase their sensitivity to subtle algorithmic modulations, including adaptive melodic shifts, personalized rhythm synchronization, and emotionally congruent auditory changes (69).

Furthermore, individuals with a stronger sense of presence are more likely to attribute these auditory changes to the intentional and responsive capacity of the AI system, rather than processing them as static or pre-programmed content (70). The subjective experience of immersion thus promotes a transition from passive auditory reception to the perception of active, reciprocal interaction. In other words, when users feel psychologically situated within the AI-generated music environment, they are more likely to perceive the system as interacting with their real-time cognitive and emotional states (71).Accordingly, this study argues that presence provides the perceptual foundation for perceived interactivity. Based on this hierarchical progression from spatial anchoring to relational attribution, this study proposes the following hypothesis:

H5: Presence significantly and positively predicts individuals’ Perceived Interactivity within the brief AI-generated music listening context.

### 2.5 Perceived interactivity and psychotic-like experiences

By enhancing social cognitive transparency, Perceived Interactivity exerts a dual inhibitory effect on Psychotic-Like Experiences (PLEs) (53). Beyond reshaping digital auditory experiences and relaxation paradigms, Perceived Interactivity establishes a dynamic emotional feedback flow that drastically heightens the internal and external transparency of human-computer interaction intentions (72). A pivotal driver of this inhibitory effect lies in the rigorous alignment between individuals’ intention and AI responses. Particularly within highly responsive digital health interactions, this alignment above yields a unique “relational bond premium” (73). Leveraging technologies such as adaptive audio algorithms and affective computing, individuals can predict real-time emotions through the musical feedback (74), thereby forging a verifiable, dynamic mapping between subjective psychological needs and external auditory compensation. Such high-fidelity emotional responses substantially diminishes the costs of intent inference for individuals with schizophrenia, by granting brain mentalizing system to access non-threatening signals without inhibitory filtering (75). Serving as a crucial informational mediator within human social cognition, the mentalizing network employs its mental navigation mechanisms to interpret the resonance cues embedded in AI music and assess the environmental inclusion (76). This process directly corrects flawed appraisals of potential hostility and social threats from the external world (77), which optimizes the transmission of secure attachment signals and elicits endogenous positive emotions, ultimately alleviating the resource depletion and fostering a more proactive, adaptive re-engagement with the real world (78).

Individuals whose “social brain” demonstrates profound trust in controllable AI interactions find their cognitive system subject to robust expectations of strong positive feedback (79). This potent constraint could effectively inhibit the “hostile attribution bias” and mitigate pathological defensive mechanisms such as paranoid delusions (51). Operating under the expectations of secure interaction, individuals are more likely to maintain the consistency between internal emotional states and external expressions, so as to optimize their social cognitive resources allocation and to transform the inherent advantages of digital relational bonding into substantive psychological recovery (80). This precise transmission mechanism greatly reduces both the frequency and the intensity of Psychotic-Like Experiences (PLEs) (81). In terms of frequency, by alleviating constraints on emotion-regulation, individuals’ brain can adaptively process normative sensory inputs (82). In terms of the intensity, enhanced experiences of positive interaction inhibit paranoid rumination, catalyzing the transition into a healthy psychological state characterized by authentic social safety and self-consistency (83). Guided by this logical reasoning, this study proposes the following hypothesis:

H6: Perceived Interactivity significantly and negatively predicts individuals’ Psychotic-Like Experiences (PLEs).

### 2.6 The moderating effect of arousal

In the present study, arousal refers specifically to emotional arousal, that is, the intensity or activation level of an individual’s affective state, ranging from calmness and relaxation to tension, anxiety, and hypervigilance (84). This concept is distinct from general physiological arousal or baseline neural activation, although emotional arousal is often accompanied by physiological responses such as autonomic activation and heightened sensory sensitivity. Drawing on the dimensional model of affect, emotional arousal is treated here as the activation dimension of emotion, independent of emotional valence. In the context of PLEs and brief AI-generated music listening context, the present study particularly focuses on high negative emotional arousal, such as anxiety, nervousness, and hypervigilance, because these states may consume cognitive resources and interfere with mindfulness-based attentional regulation (85).

Trait Mindfulness supports a core cognitive regulatory mechanism to alleviate Psychotic-Like Experiences (PLEs), but the efficiency of this top-down information processing is strictly constrained by individuals’ bottom-up physiological Arousal (86). Serving as a neural representation for the baseline level of activation maintained by central nervous system (CNS), Arousal acts as a pivotal “physiological regulatory valve” in brief AI-generated music listening contexts (87). Based on the Cognitive Resource Theory, abnormal Arousal (such as hypervigilance or severe anxiety) generates intense “systemic physiological noise” across individual’s neural substrate of sensory (Chen et al., 2024). This noise drastically drains the working memory bandwidth typically reserved for reality monitoring. Behaving on these boundary conditions, the dual facilitative effect of Mindfulness on the digital intervention experience undergoes a pronounced heterogeneous divergence (88). In particular, on the spatial dimension of transformation from Mindfulness to Presence, the neural agitation triggered by high-levels Arousal disrupts the stability required for individuals’ attention to successfully anchoring in the present moment (89). Such physiological interference in the information flow attenuates the high-fidelity mapping between auditory exposure and psychological anchoring, increases brain’s cognitive validation costs, and severely compromises the “spatial construction premium” afforded by Mindfulness (90).

From the perspective of relationship between mindfulness-induced Perceived interactivity and Mindfulness, individuals with enhanced Arousal are often accompanied by defensive survival instincts and rigid cognitive schemas (91). Such physiological hyperactivation forces the mentalizing network of brain into a state of “cognitive lockdown”. Consequently, this system struggles to fluidly capture and process the dynamic emotional feedback embedded in AI music, which hinders the transparent interpretation of digital interaction intention by mindfulness, and severely reduces the conversion efficiency of emotional predictability (92). Furthermore, based on the diathesis-stress model of psychosis, Arousal inherently acts as a potent catalyst for aberrant salience. Maintaining elevated arousal over an extended period exacerbates the information asymmetry between internal delusions and external realistic stimuli. Therefore, the resistance to intervention on Mindfulness attempts to decouple pathological rumination through “cognitive defusion” will increase exponentially (93).

Conversely, for individuals with moderate or low levels of Arousal, a quiescent physiological substrate actively reduces the frictional costs of internal information processing (94), which maximizes the self-regulatory efficacy of Mindfulness, enabling innovative digital intervention resources far more precisely into the deeper trajectories of psychological rehabilitation (95). Ultimately, as the pivotal environmental and physiological boundary condition, Arousal dynamically dictates both the efficiency and the intensify with which mindfulness transforms into substantive recovery, namely, superior experiential quality and pronounced symptom relief (96). Guided by this logical reasoning, this study proposes the following hypotheses:

H7a: Arousal moderates the relationship between Mindfulness and Presence; higher the levels of arousal, lower the positive prediction of Mindfulness on Presence.

H7b: Arousal moderates the relationship between Mindfulness and Perceived interactivity; higher the levels of Arousal, lower the positive prediction of Mindfulness on Perceived Interactivity.

H7c: Arousal moderates the relationship between Mindfulness and Psychotic-like Experiences (PLEs); the negatively suppressive impact of Mindfulness on Psychotic-like Experiences (PLEs) varies significantly across different levels of Arousal.

**Figure 1:**
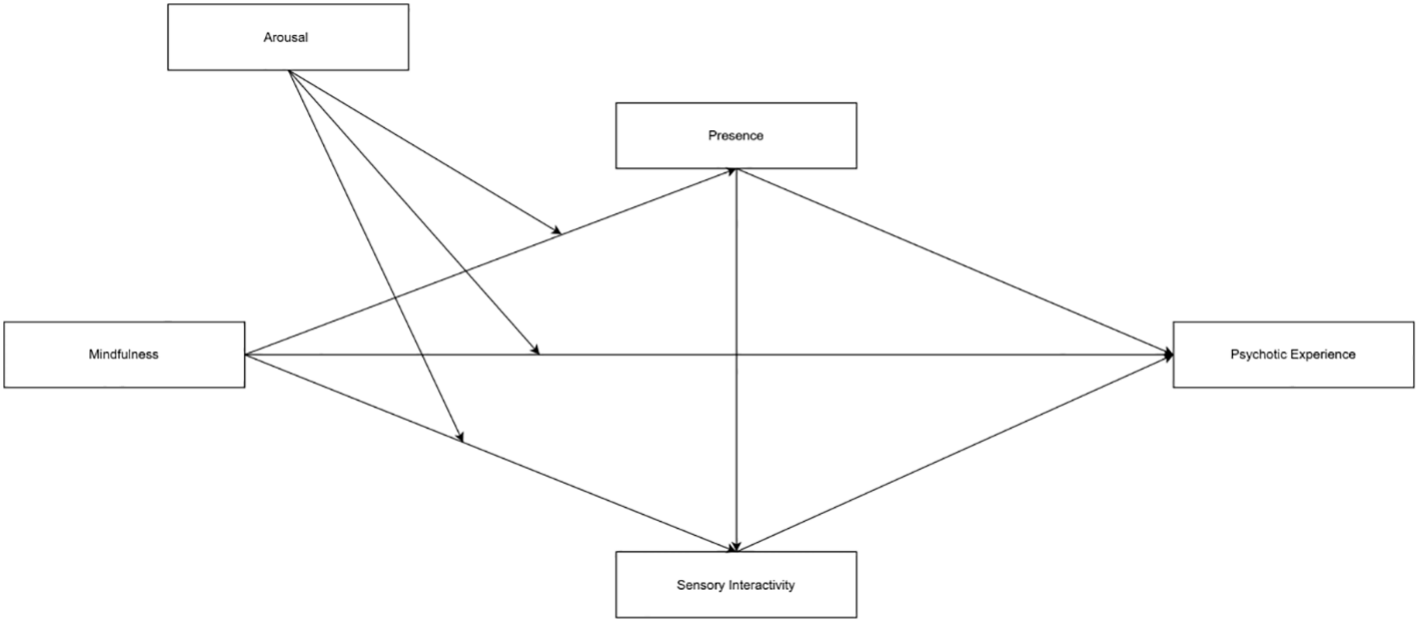
Theoretical model of this study.

The theoretical model of this study is presented in the figure below (**Figure 1**).

## 3 Methods

### 3.1 Participant recruitment and data collection procedure

This study employs a cross-sectional online questionnaire design, participants across mainland China have been recruited between October and November 2025 (97). Given that psychotic-like experiences (PLEs) exist along a psychosis continuum in the general population, young adults navigating academic and career transitions (98) tend to experience heightened psychological stress, rendering them a vulnerable group for subclinical psychological symptoms. This population also shows relatively high acceptance toward digital audio-based experiential tasks, which supports good ecological validity for research involving such digital audio stimuli. (99). Consequently, questionnaires were systematically distributed utilizing a combination of snowball sampling and targeted online survey platforms (100). To precisely capture the core digital interaction variables within the proposed model, this study innovatively adopted a standardized “experience-then-measure” data collection protocol (101). Specifically, before starting the questionnaire, all participants were asked to wear headphones in a quiet setting and listen to a roughly 3-minute piece of AI-generated music composed for mindfulness-oriented listening experience. (102). Upon finishing the audio clip, the system automatically directed participants to the questionnaire page, so that their state ratings of presence, perceived interactivity, and arousal could be captured in response to the immediately preceding digital auditory stimulus. (29). Throughout the data collection and subsequent data cleansing, the research team strictly adhered to the ethical principles delineated in the Declaration of Helsinki. Accordingly, all participants were required to review an informed consent form on the landing page and obtain informed consent before proceeding with the questionnaire.

To safeguard data validity and methodological rigor, the survey instrument incorporated multiple stringent quality-control methods. Before proceeding to the questionnaire, all participants were required to read an informed consent statement on the landing page and provide electronic informed consent. Participation was voluntary, and participants were informed that they could withdraw at any time. No personally identifiable information was collected. First, exclusion screening was employed to determine the targeted participant, filtering out individuals who self-report on severe diagnosed psychiatric disorders, such as clinical schizophrenia or profound hearing impairments, thereby ensuring all the participants are strictly represented a “non-clinical population” (103). Additionally, for those participants who failed to pass the two randomly dispersed attention-check, their responses were excluded (104). Those responses completed within 180 seconds, with repetitive or clearly patterned answers were excluded as well. To prevent sample contamination, this study strictly restricted to a single entry per IP address and device terminal to a single submission. Following this rigorous data-cleansing trajectory, a total of 527 unequivocally valid responses was retained for analysis. Regarding the final demographic profile (detailed in **Table 1**) , females marginally outnumbered males (55.8% vs. 44.2%). The sample demonstrates a noticeable young-aged tendency, with an average age of 24.83 years (SD ≈ 5.2), where the 18–25 age group accounted for the overwhelming majority at 68.7%. Participants also demonstrate a high standard of educational background, with 82.2% holding a bachelor’s degree or higher. Ultimately, both the magnitude and quality of this refined sample thoroughly satisfy the statistical power thresholds required for Structural Equation Modeling (SEM) analysis (105).

**Table 1:** Results of descriptive statistical analysis.

Variables	Categories	n	%
Gender	Male	233	44.2
	Female	294	55.8
Age	18–25 years	362	68.7
	26–35 years	138	26.2
	≥ 36 years	27	5.1
Education Background	High school or below	94	17.8
	Bachelor’s degree	286	54.3
	Master’s degree or above	147	27.9

In this study, AI-generated music refers to music produced through an artificial intelligence-based music generation system rather than music selected from existing human-composed or sample-based music libraries. The present study used AI-generated music as a listening context to examine participants’ perceived presence, perceived interactivity, emotional arousal, and their associations with trait mindfulness and PLEs. However, because the study did not include a comparison condition involving non-AI music or sample-based music, the findings should not be interpreted as evidence that AI-generated music has unique psychological effects. Instead, the results should be understood as associations observed within a brief AI-generated music listening context. Future studies should directly compare AI-generated music, sample-based music, and ordinary music listening conditions to clarify whether the observed associations are specific to AI-generated music.

### 3.2 Measures

All measurement tools adopted in this research were selected from mature and widely validated domestically and internationally. To guarantee semantic equivalence and measurement validity across cultural contexts, all original English scales underwent a rigorous back-translation process (106). Meanwhile, several item expressions were accurately revised to fit the digital intervention setting of this study. For instance, the general term “environment” was specified as “AI music experience”. To fully evaluate the reliability of the measurement model, this study simultaneously reports coefficients of Cronbach’s α, Composite Reliability (CR), and Average Variance Extracted (AVE). Detailed measurement information for each variable is presented below:

(1) Mindfulness The Five Facet Mindfulness Questionnaire (FFMQ) (107) was employed to evaluate participants’ level of Mindfulness. This scale consists of 39 items, covering five core dimensions: Observation, Description, Aware Actions, Non-Judgmental Inner Experience, and Non-Reactivity. Higher scores indicate a higher level of individuals’ mindfulness in their daily lives. In this study, the scale exhibits excellent psychometric properties, with a Cronbach’s α coefficient of 0.92, a Composite Reliability (CR) of 0.94, and an Average Variance Extracted (AVE) of 0.62, indicating excellent internal consistency and convergent validity.

(2) Presence To measure participants’ level of spatial anchoring and psychological engagement while listening to AI music, this study adapted the Presence scale (108) with 10 items (e.g., “While listening to this AI music, I felt completely immersed in the space created by the music”). Higher scores indicate a deeper presence in the digital auditory environment. Data analysis shows that the scale has good reliability and validity, with a Cronbach’s α coefficient of 0.87, a CR value of 0.89, and an AVE value of 0.68.

(3) Perceived interactivity The adapted scale was used to assess participants’ responsibility and emotional resonance of the AI music (109). Combined with the digital music context, the most representative 4 items were selected (e.g., “This AI music seems to perceive and respond dynamically to my current emotional state”). Despite the limited number of items, this scale still showed good measurement stability, with a Cronbach’s α coefficient of 0.76, a CR value of 0.79, and an AVE value of 0.61, which fully meets the statistical threshold requirements for Construct Validity (110).

(4) ArousalThis study used a state-oriented assessment tool to measure participants’ immediate physiological and emotional activation level after the intervention, referring to existing Arousal assessment dimensions (109). This scale includes 3 items, requiring participants to evaluate their immediate central nervous system activation level just after listening to AI music (e.g., “I feel mentally excited/active now”). Higher scores indicate a higher level of Arousal. Considering that this scale is a simplified version (consists of only 3 items), its Cronbach’s α coefficient (0.72) and CR value (0.74) are both within the ideal acceptable range, and the AVE value reaches 0.59, indicating sufficient convergent validity.

(5) Psychotic-like experiences (PLEs)A brief community psychopathology assessment tool was used to measure participants’ subclinical psychosis (111). This scale includes 8 items, mainly measuring the frequency and related intensity of symptoms such as paranoia, hallucinations, and depersonalization (e.g., “Have you ever felt that others are looking at you strangely or are hostile to you?”). Higher total scores indicate more frequent and intensified Psychotic-Like Experiences (). The Cronbach’s α coefficient of this dependent variable scale was 0.83, the CR value was 0.85, and the AVE value was 0.64, indicating that the measurement results are highly reliable (**Table 2**).

**Table 2:** Fornell–Larcker criterion for discriminant validity.

Variables	Mindfulness	Presence	P. interactivity	Arousal	PLEs
Mindfulness	0.78				
Presence	0.48	0.81			
P. Interactivity	0.40	0.46	0.80		
Arousal	0.24	0.30	0.28	0.76	
PLEs	-0.43	-0.36	-0.30	-0.20	0.79

### 3.3 Data analysis strategy

All statistical computations and hypothesis testing procedures were executed utilizing SPSS version 26.0 and Mplus version 8.3. Structured as an integrated analytical workflow, specific steps are as follows: First, the inherent Common Method Bias (CMB) with self-reported data has been addressed. Harman’s single-factor test in SPSS was deployed to detect potential Common Method Bias (CMB). Following this preliminary diagnostic, descriptive statistics (means and standard deviations) alongside Pearson correlation analyses were computed to meticulously delineate the bivariate associations among the focal variables. Second, a rigorous evaluation has been conducted on the reliability and validity of the measurement model. Confirmatory Factor Analysis (CFA) was conducted with Mplus, relying on goodness-of-fit indices (X^2^/df, CFI, TLI, RMSEA, and SRMR) to ascertain that the empirical data could adequately map onto the theoretical framework. Meanwhile, Composite Reliability (CR) and Average Variance Extracted (AVE) metrics were derived to estimate both the convergent validity and discriminant validity of the latent variables. Third, the main effect and proposed hypotheses have been tested. Structural Equation Modeling (SEM) was applied to investigate the direct predictive path from Mindfulness to Psychotic-Like experiences (PLEs), after which Presence and Perceived interactivity were integrated into the model as sequential mediators. To guarantee the statistical robustness of these indirect effects, a bias-corrected nonparametric percentile Bootstrap method featuring 5,000 resamples was utilized. The indirect pathways were deemed statistically significant, with their corresponding 95% confidence intervals (CIs) excluded zero. Last, the serial mediation framework was expanded to incorporate Arousal and its interaction with the predictor (Mindfulness). To systematically avoid multicollinearity issues, mean-centering was applied to all relevant continuous variables prior to constructing interaction terms. Upon detecting significant path coefficients for these interactions, simple slope analyses were plotted to empirically dissect how the Mindfulness affect both the digital intervention experience (Presence and Perceived Interactivity elicited by AI music) and Psychotic-Like Experiences diverged under high (+1 SD) and low (-1 SD) arousal, thereby comprehensively validating the proposed moderated mediation model.

## 4 Results

### 4.1 Descriptive statistical analysis

The final valid sample for analysis comprised 527 participants, with relatively balanced gender distribution: females (n=294,55.8%) slightly outnumbered males (n=233,44.2%). In terms of age, this group predominantly consists of young adults aged 18 to 25 years old (n=362,68.7%), followed by individuals aged 26 to 35 years (n=138,26.2%), whereas those aged 36 and above accounted for a very small proportion (n=27,5.1%). In terms of educational background, participants are generally well-educated. The majority held a Bachelor’s degree (n=286,54.3%), and a substantial proportion possessed a Master’s degree or higher (n=147,27.9%), with only 17.8% (n=94) reporting a high school education or below.

### 4.1 Common method bias and measurement model assessment

Given that all variable data were collected via participant self-reports, we performed rigorous tests to examine potential common method bias (CMB) before hypothesis testing, which helps mitigate concerns regarding method-specific variance in the observed correlational patterns. Frist, as a preliminary screening mechanism, Harman’s single-factor test was deployed for the exploratory factor analysis. All 64 measurement items are included in an unrotated principal component analysis. This exploratory procedure extracted four distinct factors possessing eigenvalues greater than 1.0, with the primary general factor accounting for exactly 50.05% of the total variance. Although this specific metric hovers at the extreme periphery of the conventional 50% threshold, existing methodological literature posits that the inherent conservatism of Harman’s single-factor test renders it empirically inadequate as a standalone diagnostic criterion for CMB (112). Second, this study incorporates correlation matrix inspections and Confirmatory Factor Analysis (CFA) for cross-validation. Following the criterion proposed by Pavlou et al. (2007), latent variable correlation coefficients below the 0.90 threshold may ease concerns that the dataset is substantially affected by common method bias (CMB). Third, further inspection of the correlation matrix (summarized in **Table 3**) indicates that the absolute values of all Pearson correlation coefficients between core variables remain within an acceptable range, with the largest absolute correlation considerably lower than 0.80. Collectively, this set of complementary results helps mitigate initial concerns about common method bias (CMB).

**Table 3:** Results of moderating effect analysis.

Hypothesis	Moderation path interaction term	β	p	95% CI	Decision
H7a	Emotional Arousal × Mindfulness → Presence	0.16	<.001	[0.107, 0.213]	Significant moderation, but opposite to hypothesized direction
H7b	Emotional Arousal × Mindfulness → Perceived Interactivity	0.19	<.001	[0.116, 0.264]	Significant moderation, but opposite to hypothesized direction
H7c	Emotional Arousal × Mindfulness → PLEs	−0.14	<.01	[−0.214, −0.066]	Supported

What’s more, the Confirmatory Factor Analysis (CFA) was executed with Mplus 8.3 to evaluate the benchmark five-factor measurement model, including Mindfulness, Presence, Perceived interactivity, Arousal, and Psychotic-Like Experiences (PLEs). The goodness-of-fit for this benchmark five-factor measurement model was excellent (X^2^/df = 2.34, RMSEA = 0.051, SRMR = 0.062, CFI = 0.94, TLI = 0.93), with all indices exceeding stringent statistical benchmarks. These superior multi-factorial fit statistics further confirm that the five variables maintain robust independence and discriminant validity at the latent level, and that the underlying data architecture is not dictated by a single, pervasive “ Common Method Bias (CMB)”. Synthesizing this triangulated evidence, Harman’s single-factor test, the correlation matrix inspections, and the CFA fit indices, it is obvious that the data remains devoid of substantive common method interference. Consequently, the data rigorously satisfies the foundational prerequisites for subsequent analysis of main effect, mediational, and moderating pathway estimations within the Structural equation modeling (SEM).To better verify the scientific rigor of this study, we present the Fornell–Larcker discriminant validity table for supplementary evidence.

### 4.2 Structural model and main effects analysis

Subsequent to confirming that the established measurement model possesses excellent reliability, validity, and goodness-of-fit metrics, a comprehensive Structural equation model (SEM) was constructed utilizing Mplus 8.3 to rigorously evaluate the direct pathways among the core latent variables. The analytical model was operationalized through Maximum Likelihood (ML) estimation, operating in tandem with a nonparametric percentile Bootstrap procedure featuring 5,000 resamples to compute highly robust standard errors (SE) and corresponding 95% confidence intervals (CI). As systematically delineated in **Table 4**, all direct-effect hypotheses were strongly supported by empirical data (p<0.001).

**Table 4:** Results of hypotheses on main effect paths.

Hypothesis	Structural path	β	p	95% CI	Decision
H1	Mindfulness→PLEs	-0.41	<.001	[-0.497, -0.323]	Supported
H2	Mindfulness→Presence	0.32	<.001	[0.245, 0.395]	Supported
H3	Mindfulness→Perceived Interactivity	0.28	<.001	[0.181, 0.379]	Supported
H4	Presence→ Perceived Interactivity	0.26	<.001	[0.183, 0.337]	Supported
H5	Presence→PLEs	-0.29	<.001	[-0.361, -0.219]	Supported
H6	Perceived Interactivity→PLEs	-0.22	<.001	[-0.313, -0.127]	Supported

First, path analysis results showed a strong, statistically significant negative correlational association between trait mindfulness and psychotic-like experiences (PLEs) [β = −0.41, p < 0.001, 95% CI (−0.497, −0.323)]. This pattern is consistent with Hypothesis 1, suggesting that dispositional mindfulness may correspond to lower levels of subclinical psychopathological symptoms within this sample. Second, when examining correlational patterns linked to brief AI-generated music listening experiences, path analyses indicated that trait mindfulness was significantly and positively correlated with presence [β=0.3, p<0.001, 95%CI(0.245,0.395)] and perceived interactivity [β=0.28, p<0.001, 95%CI(0.181,0.379)]. These observed patterns are consistent with Hypotheses 2 and 3, suggesting that individuals reporting higher mindfulness tended to report stronger senses of spatial anchoring and emotional resonance within this digital auditory setting in the current sample. Third, when examining sequential associations between the two digital experience variables, the dataset indicated a sequential correlational pattern between these two constructs: presence was significantly and positively correlated with perceived interactivity [β=0.26, p<0.001, 95% CI(0.183,0.337)]. This observed pattern provides preliminary statistical support for the proposed relational sequence in which a sense of spatial anchoring may coincide with greater interactive resonance within this AI music listening scenario. Last, when examining correlational patterns linking these digital experiential variables to psychotic-like experiences (PLEs), presence [β=−0.29, p<0.001, 95%C(−0.361,−0.219)] and perceived interactivity [β=−0.22, p<0.001, 95%CI(−0.313,−0.127)] both showed statistically significant negative correlations with PLEs in this sample. It implies that enhanced spatial anchoring and deeper emotional engagement with the AI system could be transformed into a more efficacious detachment from Psychotic-Like Experience, such as paranoia and derealization. Collectively, those highly significant direct pathways establish a solid foundation for further analysis on serial mediation and moderated mediation.

### 4.3 Moderating effect analysis

To examine the boundary conditions of the associations between trait mindfulness and AI music-related outcomes, this study tested the moderating role of emotional arousal. Specifically, we examined whether emotional arousal moderated the relationships between trait mindfulness and three key outcomes: presence, perceived interactivity, and psychotic-like experiences (PLEs). Before creating the interaction terms, both trait mindfulness and emotional arousal were mean-centered to reduce potential multicollinearity. The interaction term was then entered into the structural model, and the significance of the moderation effects was examined using the nonparametric percentile bootstrap procedure with 5,000 resamples.

The results showed that emotional arousal significantly moderated several pathways in the model. However, the direction of some moderation effects was not consistent with our original expectations. Therefore, these findings should be interpreted cautiously, particularly given the cross-sectional and self-report nature of the present study.

First, for the pathways related to digital interaction experience, the interaction term between emotional arousal and trait mindfulness had a significant positive association with presence [β = 0.16, p <.001, 95% CI (0.107, 0.213)] and perceived interactivity [β = 0.19, p <.001, 95% CI (0.116, 0.264)]. These results indicate that emotional arousal significantly moderated the relationships between trait mindfulness and both presence and perceived interactivity. However, the direction of the interaction was opposite to the original hypotheses. H7a and H7b originally assumed that higher emotional arousal would weaken the positive associations between trait mindfulness and these digital experience variables, because high arousal was expected to consume attentional and cognitive resources. Contrary to this expectation, the positive interaction coefficients suggest that the positive associations of trait mindfulness with presence and perceived interactivity were stronger under higher levels of emotional arousal.

This unexpected pattern requires cautious interpretation. Our original hypotheses were based on the cognitive-load perspective, assuming that higher emotional arousal would consume attentional resources and weaken the associations between trait mindfulness and digital experience outcomes. However, in the AI music context, emotional arousal may not function only as a disruptive burden. Because music is inherently affective and sensory, higher emotional arousal may increase the salience of auditory cues, strengthen emotional engagement, and make changes in AI-generated music more noticeable. For individuals with higher trait mindfulness, who tend to maintain receptive and non-reactive attention to present-moment experiences, such arousal may help them attend more closely to musical details and system feedback. This may explain why the associations of trait mindfulness with presence and perceived interactivity became stronger, rather than weaker, under higher emotional arousal. Nevertheless, this interpretation remains tentative. The arousal measure may have captured general emotional activation or engagement rather than purely negative arousal, and the non-clinical/subclinical sample may not have experienced arousal levels high enough to impair attentional control. Therefore, H7a and H7b were supported only in terms of significant moderation effects, whereas the hypothesized weakening direction was not supported. Future studies should further examine this issue with more refined arousal measures and longitudinal or experimental designs.

This result may also be related to the specific characteristics of AI-generated music. Unlike sample-based music, which usually relies on preselected and relatively fixed musical materials, AI-generated music can produce more adaptive, dynamic, and context-sensitive auditory patterns. Such generative features may make emotional arousal more likely to be experienced as engagement, novelty, and responsiveness rather than as cognitive overload. Therefore, in this study context, higher emotional arousal may have amplified users’ sensitivity to the immersive and interactive qualities of AI music, thereby strengthening the associations between trait mindfulness and both presence and perceived interactivity.

Second, for the pathway related to subclinical symptomatology, the interaction term between emotional arousal and trait mindfulness had a significant negative association with PLEs (β = −0.14, p <.01, 95% CI [−0.214, −0.066]). Given that the main association between trait mindfulness and PLEs was negative, this significant negative interaction indicates that the negative association between trait mindfulness and PLEs became stronger when emotional arousal was higher. In other words, individuals with higher trait mindfulness tended to report fewer PLEs, and this association was more pronounced among those with higher emotional arousal. This finding is consistent with H7c, which proposed that the relationship between trait mindfulness and PLEs would vary across different levels of emotional arousal.

However, this result should also be interpreted as an association rather than evidence of a causal buffering mechanism. Although the pattern is consistent with the possibility that mindfulness may be more strongly related to lower PLEs under higher emotional arousal, the present study cannot determine whether mindfulness reduces PLEs, whether lower PLEs facilitate mindful awareness, or whether other unmeasured variables contribute to this association. Therefore, H7c was supported statistically, but causal conclusions should not be drawn from this result.

**Figure 2:**
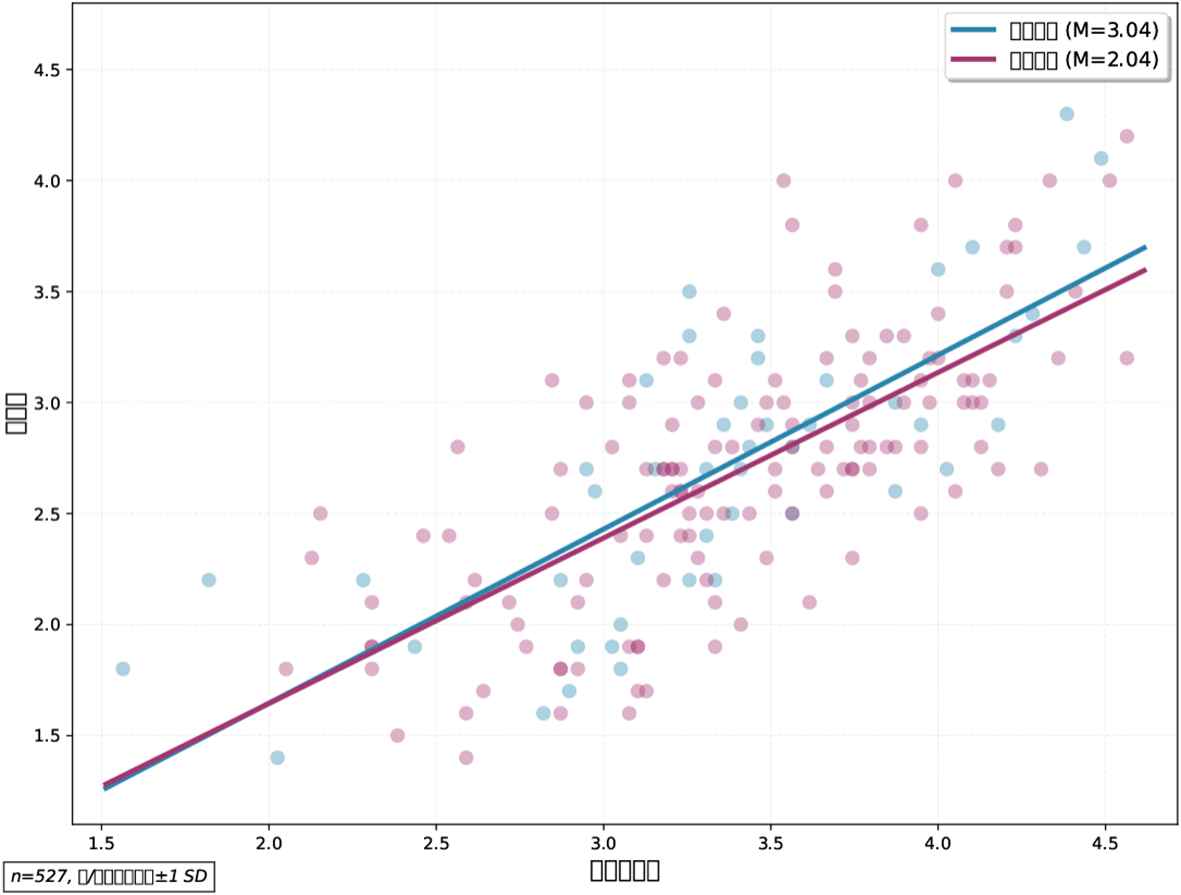
Plot of moderating effect in H6a.

**Figure 3:**
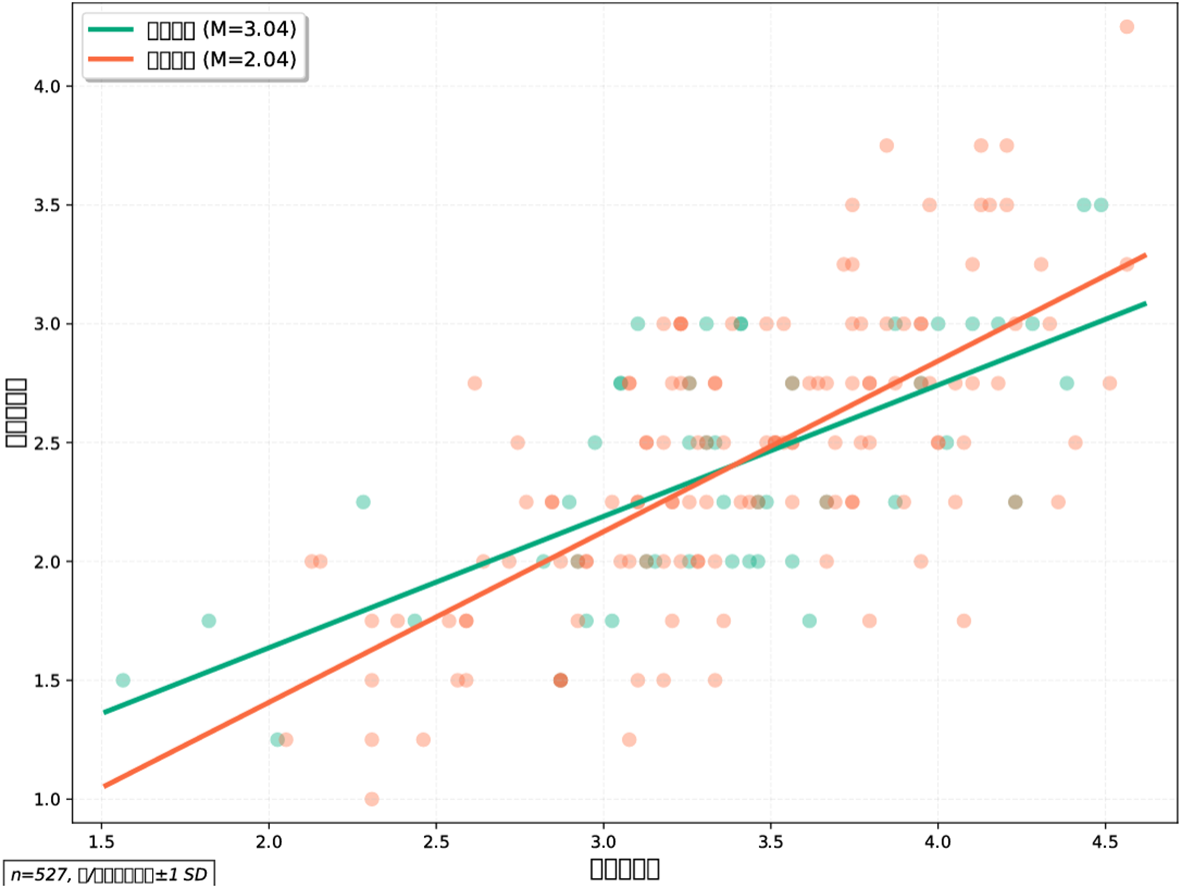
Plot of moderating effect in H6b.

**Figure 4:**
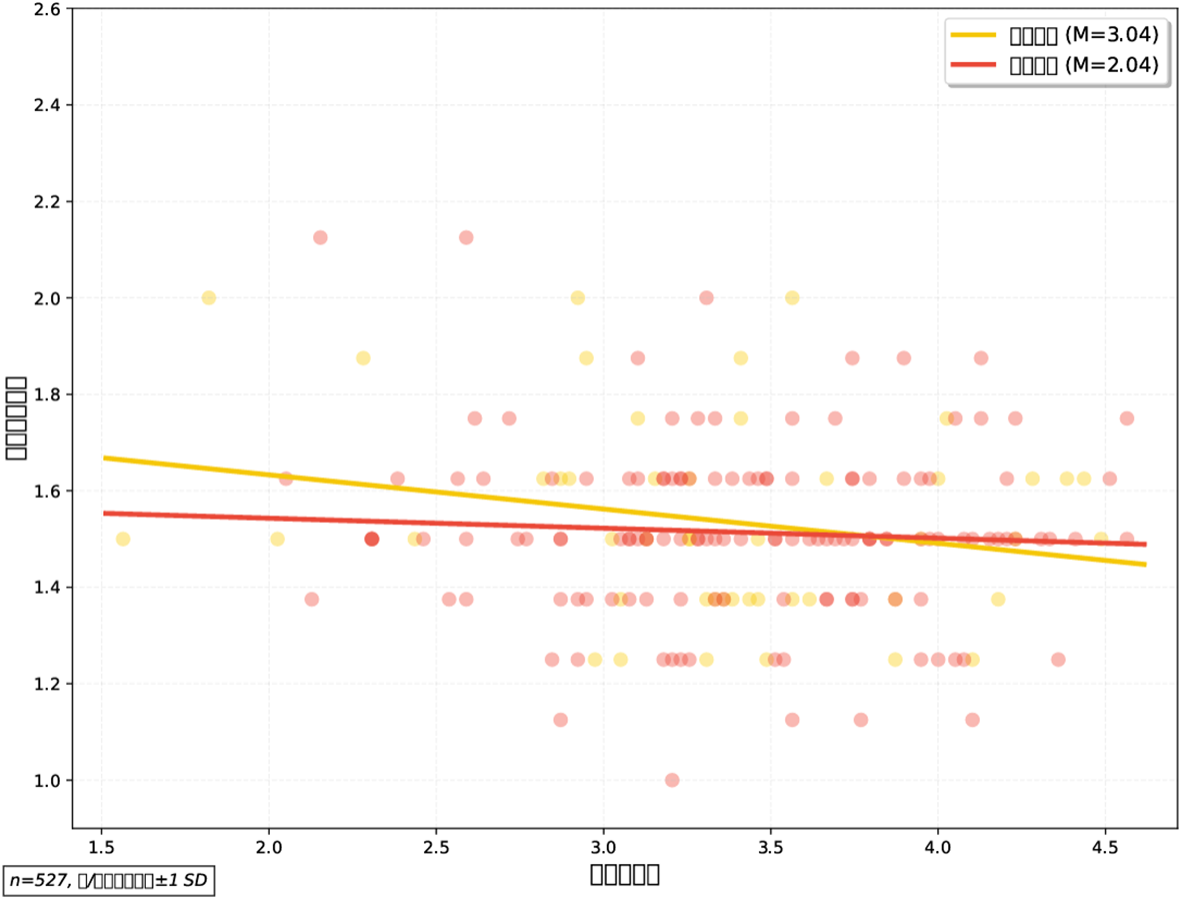
Plot of moderating effect in H6c.

**Figure 5:**
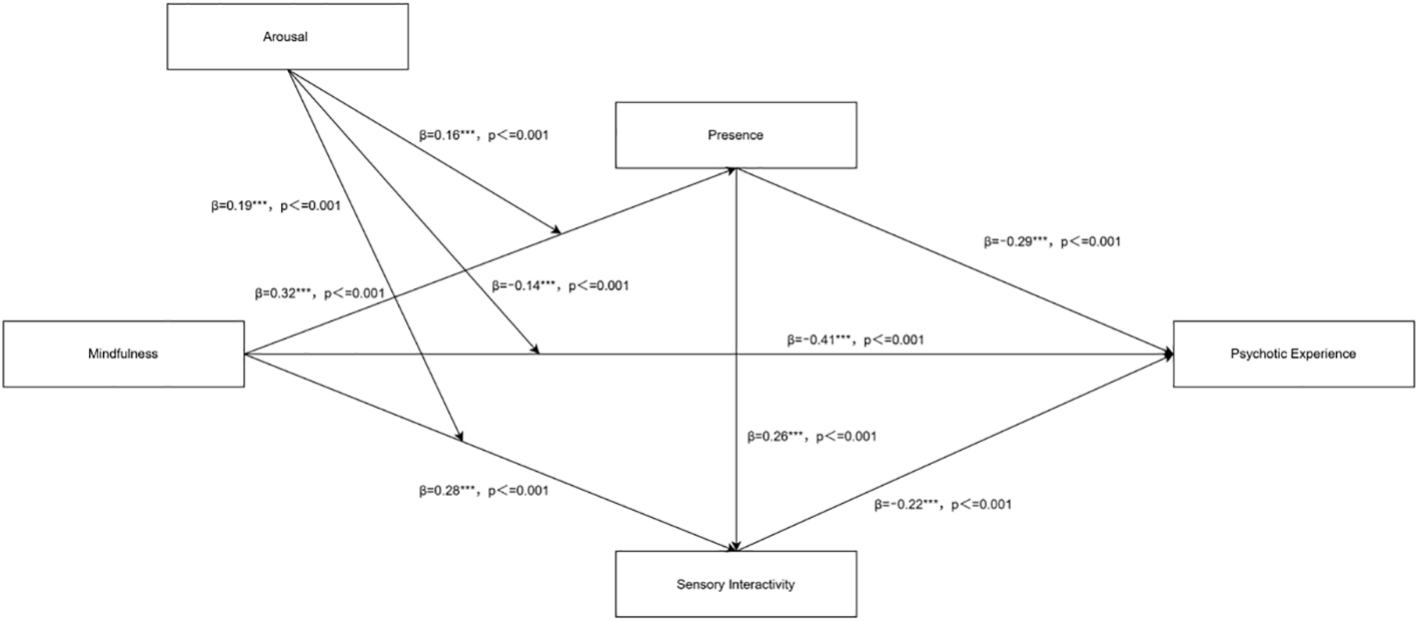
Results of structural equation modeling test. *** represents statistical significance at the level of p < 0.001, indicating extremely significant statistical differences between the tested variables.

To further illustrate these moderation effects, simple slope analyses were conducted. The associations between trait mindfulness and presence, perceived interactivity, and PLEs were plotted separately at high (+1 SD) and low (−1 SD) levels of emotional arousal, as shown in **Figures 2**–**4**. These plots provide a visual representation of how the strength of the associations between trait mindfulness and the outcome variables differed across levels of emotional arousal. Finally, the full structural model, including all tested paths and significant interaction effects, is presented in **Figure 5**.

## 5 Discussion

### 5.1 Summary of main findings

Importantly, the present study should be understood as examining psychological associations within a brief AI-generated music listening context, rather than as testing the clinical efficacy of an brief AI-generated music listening context (113). Given the three-minute exposure and the absence of a comparison group, the findings cannot determine whether the observed associations are specific to AI-generated music or reflect broader effects of music listening. Grounded in digital mental health, human-computer interaction, and abnormal psychology, this study examined the associations among trait mindfulness, psychotic-like experiences (PLEs), presence, perceived interactivity, and emotional arousal in the context of AI music use (81, 114). The results showed that trait mindfulness was negatively associated with PLEs, supporting H1 (115, 116). This finding is consistent with previous research suggesting that mindfulness may be related to better attentional regulation, reduced cognitive reactivity, and lower vulnerability to psychotic-like symptoms.

In addition, trait mindfulness was positively associated with both presence and perceived interactivity, supporting H2 and H3 (117, 118). Presence and perceived interactivity were also negatively associated with PLEs, supporting H4 and H5 (119, 120). The mediation results further indicated that presence and perceived interactivity statistically mediated the association between trait mindfulness and PLEs, supporting H6a and H6b. These findings suggest that immersive and interactive experiences in AI music contexts may be relevant to the relationship between mindfulness and PLEs.

Finally, emotional arousal moderated several associations in the model. The moderation effects on the mindfulness–presence and mindfulness–perceived interactivity links were significant, but the direction was opposite to the original hypotheses, supporting H7a and H7b only in terms of the existence of significant moderation effects. Emotional arousal also strengthened the negative association between mindfulness and PLEs, supporting H7c (121). Given that the sample mainly consisted of young and relatively highly educated participants from a single research context, these findings should be interpreted as context-specific associations within a brief AI music listening setting rather than as broadly generalizable evidence for clinical or real-world intervention effects.

### 5.2 Theoretical contributions and implications

Building on the interdisciplinary intersection between abnormal psychology and human-computer interaction, this study provides preliminary and context-specific evidence for understanding how trait mindfulness is associated with psychotic-like experiences (PLEs) in an AI music listening context (122). Rather than establishing a causal intervention mechanism, the present findings should be understood as cross-sectional associations among mindfulness, presence, perceived interactivity, emotional arousal, and PLEs. Within these boundaries, the study offers three theoretical contributions.

First, this study extends the discussion of mindfulness in PLE-related research by linking trait mindfulness with subjective experiences of digital interaction. Previous studies have mainly examined mindfulness in relation to internal cognitive and emotional processes, such as cognitive reappraisal, emotional acceptance, and reduced rumination (123). The present findings suggest that trait mindfulness is not only negatively associated with PLEs, but also positively associated with presence and perceived interactivity in an AI music context (29). This result provides preliminary evidence that mindfulness may be relevant to how individuals perceive and engage with digital environments (51). However, given the cross-sectional design, this study cannot determine whether mindfulness improves digital interaction quality, whether better digital experiences are associated with higher mindful awareness, or whether both are influenced by other unmeasured factors. Therefore, the contribution of this study lies in identifying a theoretically meaningful association between mindfulness and AI music-related experience, rather than confirming a causal pathway of digital therapeutic sensitivity (48, 124).

Second, this study contributes to the literature on human-computer interaction and digital mental health by examining presence and perceived interactivity as potential explanatory variables linking trait mindfulness and PLEs. Existing HCI research has often discussed immersion and interactivity as important but relatively separate components of user experience (125). In the present study, both presence and perceived interactivity were negatively associated with PLEs and statistically mediated the association between mindfulness and PLEs (126). These findings suggest that immersive and interactive experiences may be relevant psychological correlates in AI music-based digital mental health contexts. Specifically, presence may reflect the extent to which individuals feel experientially situated within the AI music environment, while perceived interactivity may reflect their subjective sense that the system is responsive, personalized, and emotionally engaging (98, 127). Nevertheless, these mediation results should not be interpreted as evidence of a confirmed transmission mechanism. Because the data are cross-sectional and based on self-report, the temporal order among mindfulness, presence, perceived interactivity, and PLEs remains uncertain. Thus, this study offers a tentative framework for future research, rather than a definitive explanation of how AI music experiences influence PLEs (128).

Third, the moderation findings provide a cautious theoretical refinement regarding the role of emotional arousal. Traditional cognitive load perspectives often suggest that higher arousal may consume attentional resources and weaken top-down regulatory processes (15). However, in this study, emotional arousal strengthened the associations between trait mindfulness and both presence and perceived interactivity, which was opposite to the originally hypothesized direction. This unexpected pattern suggests that, in a brief AI music listening context, emotional arousal may not necessarily function only as a cognitive burden. For mindful individuals, a certain level of emotional activation may be associated with greater sensory engagement, attentional involvement, and perceived responsiveness to the AI music environment (3, 129). However, this interpretation should remain tentative. The arousal measure used in this study may have captured a broad form of emotional activation rather than clearly distinguishing positive from negative arousal, and the young, relatively highly educated, non-clinical sample may not have experienced arousal levels high enough to impair attentional control. Therefore, this finding should be viewed as an exploratory indication that emotional arousal may have more complex or potentially nonlinear associations with digital experience, rather than as evidence of a theoretical breakthrough or causal amplification effect (130).

Overall, the theoretical value of this study lies in proposing a bounded and empirically grounded framework for examining the associations among mindfulness, AI music-related experience, emotional arousal, and PLEs. These contributions should be interpreted within the specific context of a short AI music listening experience among mostly young and highly educated non-clinical participants. Future longitudinal, experimental, and clinically oriented studies are needed to test whether the proposed associations hold across more diverse populations, longer-term AI music use, and clinically relevant settings.

### 5.3 Practical implications

The findings provide several cautious design implications for AI-generated music applications in digital mental health contexts. Because this study used a cross-sectional design, a brief listening session, and a non-clinical sample, these implications should be understood as preliminary design considerations rather than clinical recommendations or evidence of intervention efficacy (131).

First, trait mindfulness may be a relevant user characteristic when designing AI-generated music experiences. The results showed that individuals with higher trait mindfulness reported stronger presence and perceived interactivity. This suggests that AI music systems may benefit from offering optional mindfulness-oriented guidance, such as brief attention-focusing instructions before listening. However, this should be treated as a design possibility rather than evidence that mindfulness training improves responses to AI music.

Second, the findings suggest that presence and perceived interactivity may be important experiential dimensions in AI-generated music design (132). Developers may consider creating auditory environments that support immersion, clarity, and perceived responsiveness. However, the present study cannot determine whether these features reduce PLEs or produce mental health benefits. Future experimental studies are needed to test whether specific design features of AI-generated music influence users’ psychological outcomes (133).

Third, emotional arousal may be an important contextual factor in AI-generated music experiences. In this study, higher emotional arousal strengthened the associations between mindfulness and both presence and perceived interactivity. This does not mean that high arousal is an optimal timing condition for intervention. Rather, it suggests that users’ momentary emotional activation may be relevant when designing adaptive listening experiences. Future studies should examine whether self-reported, behavioral, or physiological indicators of arousal can improve personalization in AI music systems.

Overall, the practical implications of this study are exploratory. The findings may inform future design hypotheses, but they should not be interpreted as evidence for clinical effectiveness, therapeutic mechanisms, or real-world digital intervention outcomes.

### 5.4 Limitations and future directions

Notwithstanding its theoretical and empirical insights, this study has several limitations. First, the cross-sectional design precludes causal inference among trait mindfulness, presence, perceived interactivity, emotional arousal, and psychotic-like experiences (PLEs), even though the structural model showed acceptable fit indices (114). Future studies should use longitudinal, experimental, or ecological momentary assessment designs to examine temporal and causal relationships (134).

Second, all variables were measured through immediate self-report after a brief AI-generated music listening session, which may introduce transient-state effects, demand characteristics, and common method bias (Chen et al., 2024). Although Harman’s single-factor test did not indicate a dominant single factor, this evidence is limited and cannot fully rule out shared method variance. Future research should use multi-source, time-lagged, behavioral, or physiological measures, as well as more rigorous statistical controls.

Third, emotional arousal and perceived interactivity were measured with relatively few items. Thus, they may not fully capture the multidimensional nature of these constructs, including arousal intensity and valence, or interactivity features such as responsiveness, controllability, personalization, and feedback. Findings involving these variables, particularly the moderating effects of arousal and the mediating role of perceived interactivity, should therefore be interpreted cautiously.

Fourth, the study does not allow strong claims about AI-generated music as an intervention. Participants listened to AI-generated music for only three minutes, and no pre-post assessment or comparison group was included. Therefore, the findings cannot determine whether the observed associations were attributable specifically to AI-generated music, music listening in general, or other contextual factors. Future studies should include longer or repeated exposure and comparison conditions such as non-AI music, silence, or standard music listening.

Fifth, the sample consisted mainly of young, relatively highly educated, non-clinical participants from a single research context. Accordingly, the findings should be viewed as context-specific and should not be generalized directly to clinical populations or broader real-world applications (135). Future research should validate this model in more diverse samples, including ultra-high-risk or prodromal groups (136).

Finally, the unexpected positive moderating effects of emotional arousal should be interpreted cautiously. They may reflect broad arousal measurement, sample characteristics, or a nonlinear pattern in which moderate arousal facilitates engagement whereas excessive arousal becomes disruptive. Future studies should examine different types and levels of arousal using longitudinal, experimental, or physiological methods.

## Data Availability

The raw data supporting the conclusions of this article will be made available by the authors, without undue reservation.
